# Supplementary figures and images for: Assisted sexual coral recruits show high thermal tolerance to the 2023 Caribbean mass bleaching event
Source: PLoS One. 2024 Sep 18;19(9):e0309719. doi: 10.1371/journal.pone.0309719 (PMC11410220; doi:10.1371/journal.pone.0309719)

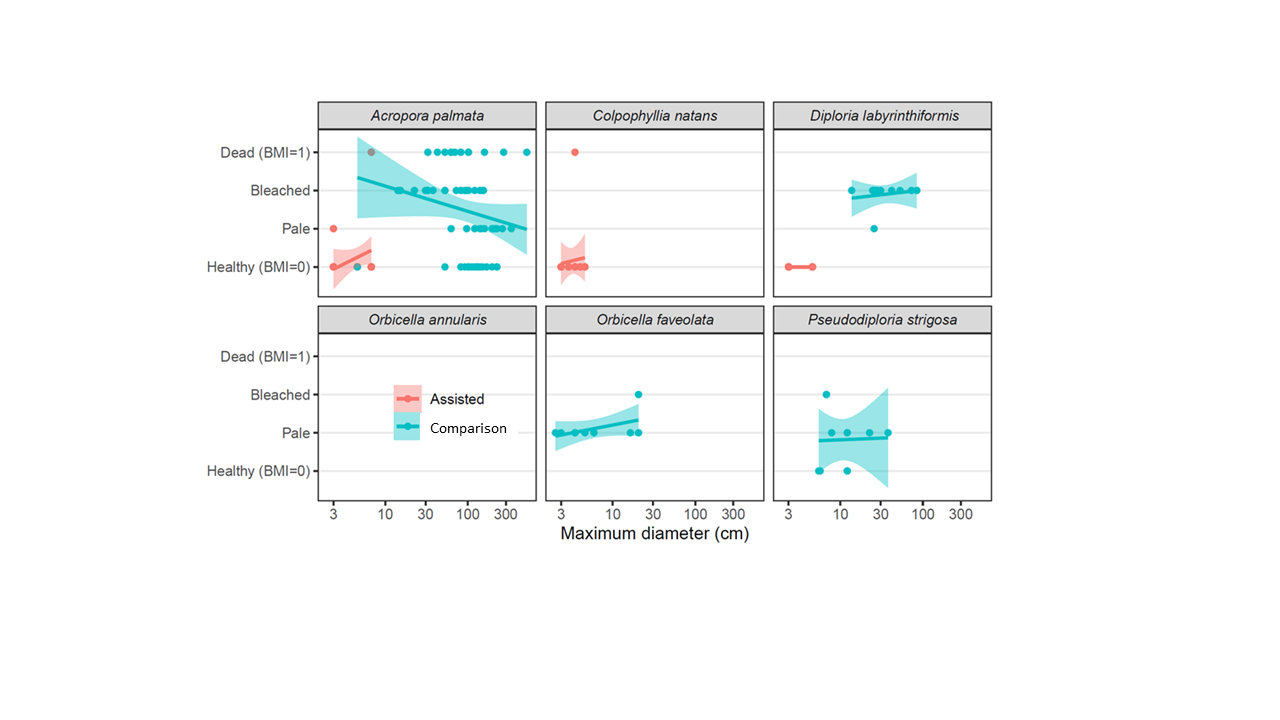

Supplement: S1 Fig — Note that colony size was not consistently recorded across the data set. (TIF) [file pone.0309719.s001.tif]
