# Supplementary material for: Assisted sexual coral recruits show high thermal tolerance to the 2023 Caribbean mass bleaching event
Source: PLoS One. 2024 Sep 18;19(9):e0309719. doi: 10.1371/journal.pone.0309719 (PMC11410220; doi:10.1371/journal.pone.0309719)
Supplement: S1 Table — The cumulative link model (CLM) uses ordinal health status scores as the response variable, the GLM and GLMM use the Bleaching and Mortality Index as the response variable, and the GLMM in addition accounts for variations in response due to region, site, or population type (proxy of census method). (DOCX) [file pone.0309719.s003.docx]

| **Species** | **Model** | **DF** | **Estimate** | **SE** | **Test statistic** | **P value** |
| --- | --- | --- | --- | --- | --- | --- |
| ***Acropora*** | CLM | NA | -1.41 | 0.16 | -8.74 | **<0.001** |
| ***palmata*** | GLM | 1058 | -0.94 | 0.17 | -5.58 | **<0.001** |
|  | GLMM | 1055 | -0.58 | 0.25 | -2.3 | **0.022** |
| ***Colpophyllia*** | CLM | NA | -2.23 | 0.6 | -3.72 | **<0.001** |
| ***natans*** | GLM | 1058 | -1.54 | 0.64 | -2.42 | **0.016** |
|  | GLMM | 1055 | -1.54 | 0.66 | -2.31 | **0.021** |
| ***Diploria*** | CLM | NA | -2.81 | 0.21 | -13.5 | **<0.001** |
| ***labyrinthiformis*** | GLM | 1058 | -2.54 | 0.3 | -8.46 | **<0.001** |
|  | GLMM | 1055 | -2.59 | 0.39 | -6.65 | **<0.001** |
| ***Orbicella*** | CLM | NA | -1.22 | 0.18 | -6.97 | **<0.001** |
| ***annularis*** | GLM | 1058 | -0.92 | 0.22 | -4.2 | **<0.001** |
|  | GLMM | 1055 | -0.93 | 0.34 | -2.75 | **0.006** |
| ***Orbicella*** | CLM | NA | -0.91 | 0.34 | -2.71 | **0.007** |
| ***faveolata*** | GLM | 1058 | -0.4 | 0.34 | -1.16 | 0.245 |
|  | GLMM | 1055 | -0.39 | 0.41 | -0.93 | 0.353 |
| ***Pseudodiploria*** | CLM | NA | -1.35 | 0.22 | -6.26 | **<0.001** |
| ***strigosa*** | GLM | 1058 | -1.21 | 0.3 | -3.99 | **<0.001** |
|  | GLMM | 1055 | -1.25 | 0.33 | -3.74 | **<0.001** |

Table S1. Model results testing for species-specific differences in bleaching responses between assisted recruits and other comparison colonies (model form: response ~ AR-Comparison * Species), such that a negative estimate is a worse health status. The cumulative link model (CLM) uses ordinal health status scores as the response variable, the GLM and GLMM use the Bleaching and Mortality Index as the response variable, and the GLMM in addition accounts for variations in response due to region, site, or population type (proxy of census method).
