# Supplementary material for: Assisted sexual coral recruits show high thermal tolerance to the 2023 Caribbean mass bleaching event
Source: PLoS One. 2024 Sep 18;19(9):e0309719. doi: 10.1371/journal.pone.0309719 (PMC11410220; doi:10.1371/journal.pone.0309719)
Supplement: Attachment — Submitted filename: ReviewResponse.pdf [file pone.0309719.s005.pdf]

secore international
giving coral reefs a future

14 Aug 2024

Dear PLoS One Editor,

We have completed corrections as requested, including Ln 121 (adding possessive for NOAA) and correcting several of the citations (italics, case of title, and journal abbreviations), as well as a couple of other proof-type corrections for typos.

Thank you for your consideration and assistance in this publishing process,

Margaret W. Miller (for co-authors)

SECORE International
2103 Coral Way, 2nd Floor
Miami FL 33145 USA

www.secore.org
info@secore.org

Margaret Miller
Research Director
tel: 305.479.3386
m.miller@secore.org
